# Perceptions of committed offenses among forensic psychiatric patients: Retrospective cross-sectional observational study

**DOI:** 10.1192/j.eurpsy.2026.10946

**Published:** 2026-07-31

**Authors:** N. Sutara, G. Arbanas, N. Buzina

**Affiliations:** 1 University Psychiatric Hospital Vrapče, Zagreb; 2 The Faculty of Medicine of the University of Rijeka, Rijeka; 3 Faculty of Croatian Studies, Zagreb, Croatia

## Abstract

**Introduction:**

Forensic psychiatry is an interdisciplinary field that combines psychiatry, medicine, and law. It treats patients with severe mental disorders who have committed criminal offenses and integrates therapeutic care with legal responsibilities. Understanding how these patients perceive their crimes is essential for effective treatment, risk management, and rehabilitation.

**Objectives:**

The aim of this study was to analyze how forensic psychiatric patients currently perceive their committed crimes, and to investigate how these attitudes are associated with the type of offense, psychiatric diagnosis, treatment history, and duration of hospitalization.

**Methods:**

The study included all forensic psychiatric inpatients at the Department of Forensic Psychiatry “Dr. Vlado Jukić”, Psychiatric Hospital Vrapče, Zagreb. Data on sociodemographics, diagnoses, treatment history, and criminal code articles were collected, with focus on patients’ most recent statements about their crimes: *acknowledges crime as part of illness*, *acknowledges crime but does not think it was due to the illness*, *amnesia*, and *denial.* Statistical analyses included descriptive summaries, cross-tabulations, Fisher’s exact tests, and non-parametric tests for group comparisons.

**Results:**

The study included 71 forensic psychiatric patients (59 men, 12 women). Most were diagnosed with schizophrenia spectrum disorders (84.5%) while smaller groups had organic, affective, substance use, or other diagnoses. Regarding attitudes toward their crimes, 40.8% acknowledged without linking to illness, 32.4% denied, 19.7% acknowledged as illness-related, and 7.0% reported amnesia. Violent offenses dominated, with threats (33.8%) and homicide (22.5%) accounting for over half of cases. A significant association was found between crime type and crime attitude (p=0.0048): homicide was more often seen as illness-related, while threats were acknowledged without illness. No significant link was found between diagnosis and attitude. There was no significant association between diagnosis and attitude (p=0.410), although descriptively, patients with organic disorders acknowledged the crime without linking it to illness, while patients with schizophrenia spectrum disorders expressed all four attitudes.

**Conclusions:**

This study shows that forensic psychiatric patients differ in how they perceive their crimes. Some acknowledge the offense as part of their illness while others admit it without linking it to illness, deny it altogether, or report amnesia. These attitudes were significantly associated with the type of offense, which suggests that patients’ subjective views could be shaped by the nature of the crime itself. Understanding these perspectives is important for clinical management, risk assessment, and rehabilitation planning in forensic psychiatry.

**Disclosure of Interest:**

None Declared

